# Phenotypic Characterization and Comparative Genomic Analysis of Novel *Salmonella* Bacteriophages Isolated from a Tropical Rainforest

**DOI:** 10.3390/ijms24043678

**Published:** 2023-02-12

**Authors:** Prasanna Mutusamy, Kirnpal Kaur Banga Singh, Lee Su Yin, Bent Petersen, Thomas Sicheritz-Ponten, Martha R. J. Clokie, Stella Loke, Andrew Millard, Sivachandran Parimannan, Heera Rajandas

**Affiliations:** 1 Centre of Excellence for Omics-Driven Computational Biodiscovery (COMBio), AIMST University, Bedong 08100, Kedah, Malaysia; 2 Department of Medical Microbiology and Parasitology, School of Medical Sciences, Health Campus, Universiti Sains Malaysia, 16150 Kota Bharu, Kelantan, Malaysia; 3 Department of Biotechnology, Faculty of Applied Sciences, AIMST University, Semeling 08100, Kedah, Malaysia; 4 Center for Evolutionary Hologenomics, Globe Institute, University of Copenhagen, Øster Farimagsgade 5, 1353 Copenhagen, Denmark; 5 Department of Genetics and Genome Biology, University of Leicester, Leicester LE1 7RH, UK; 6 Charles River Laboratories Australia Pty Ltd., Melbourne, VIC 3137, Australia; 7 School of Life and Environmental Sciences, Faculty of Science, Engineering and Built Environment, Waurn Ponds Campus, Deakin University, Geelong, VIC 3216, Australia

**Keywords:** *Salmonella*, bacteriophages, tropical rainforest, comparative genomics

## Abstract

*Salmonella* infections across the globe are becoming more challenging to control due to the emergence of multidrug-resistant (MDR) strains. Lytic phages may be suitable alternatives for treating these multidrug-resistant *Salmonella* infections. Most *Salmonella* phages to date were collected from human-impacted environments. To further explore the *Salmonella* phage space, and to potentially identify phages with novel characteristics, we characterized *Salmonella*-specific phages isolated from the Penang National Park, a conserved rainforest. Four phages with a broad lytic spectrum (kills >5 *Salmonella* serovars) were further characterized; they have isometric heads and cone-shaped tails, and genomes of ~39,900 bp, encoding 49 CDSs. As the genomes share a <95% sequence similarity to known genomes, the phages were classified as a new species within the genus *Kayfunavirus*. Interestingly, the phages displayed obvious differences in their lytic spectrum and pH stability, despite having a high sequence similarity (~99% ANI). Subsequent analysis revealed that the phages differed in the nucleotide sequence in the tail spike proteins, tail tubular proteins, and portal proteins, suggesting that the SNPs were responsible for their differing phenotypes. Our findings highlight the diversity of novel *Salmonella* bacteriophages from rainforest regions, which can be explored as an antimicrobial agent against MDR-*Salmonella* strains.

## 1. Introduction

*Salmonella* infections are a serious public health concern, especially in developing countries, where they are the main cause of morbidity and mortality [1]. These bacteria cause a variety of infectious diseases in both humans and animals, which range clinically from the common *Salmonella* gastroenteritis to life-threatening enteric fevers [2]. Enteric fever remains a major global health concern, and an estimated 9.9 to 24.2 million cases are reported annually [3]. Typhoidal *Salmonella* involves person-to-person transmission, and it can cause severe infections often requiring antibiotic treatment. In contrast, non-typhoidal salmonellosis (NTS) represents the majority of *Salmonella* infections in humans [4]. The number of reported human salmonellosis incidents was estimated to be 550 million annually, including 220 million children under the age of five [5]. It is thought that 75% of human salmonellosis is caused by contaminated food products derived from beef, pork, poultry, and eggs [6]. In recent years, *Salmonella* outbreaks have also been associated with a higher degree of consumption of fresh fruits and vegetables [7].

Antibiotics are the primary method for controlling *Salmonella* infections. However, in recent years, there have been concerns over multidrug-resistant (MDR) bacteria and the appearance of drug residues in food animals [8]. Antimicrobial resistance (AMR) is expected to cost the world economy an estimated $100 trillion annually, and it has the risk of killing 10 million people a year. By 2050, AMR could be a more serious problem than cancer, unless some actions are taken, as 4.95 M deaths were recorded in 2019 due to bacterial AMR [9]. These issues have given rise to a renewed interest in bacteriophage therapy as an alternative antimicrobial, to address the impending crisis. 

Bacteriophages are bacterial viruses that were discovered approximately 100 years ago [10]. They are the most abundant biological entities on earth, with an estimated 10^31^ phages worldwide [11]. The ability of bacteriophages to replicate and to lyse pathogenic bacteria suggests that they could play a vital role in controlling bacterial contamination in the food industry, as well as in clinical practices. To date, various commercial phage products have been developed, including Ecoshield^TM^, List-Shield^TM^, ListexP100, Omnilytics^TM^, and SalmoFresh^TM^. These products have been approved by the U.S. Food and Drug Administration (FDA) as natural food preservatives to prevent foodborne bacterial diseases [12]. Due to their efficiency, many bacteriophages infecting *Salmonella* have been isolated and are proposed as alternative biocontrol agents against different *Salmonella* serovars [13,14,15].

Most of the *Salmonella*-specific phages studied to date have come from a narrow set of environments derived from humans, such as sewage and river water [16,17,18]. Several studies have shown that new geographical locations reveal a novel diversity of phages [19,20]. As tropical rainforests are known to support a great diversity of plant and animal species, they also provide a diverse set of niches for bacteria to inhabit. With this may come novel bacterial species; the hosts of the phages, and they are likely to reveal a whole new set of phages that could potentially be developed as antimicrobials. However, despite being home to some of the oldest rainforests in the world, no studies have been performed to explore the diversity of phages that are present within the Malaysian tropical rainforests. With this in mind, we performed a pilot study to isolate and to characterize lytic bacteriophages against *Salmonella enterica* from a pristine tropical rainforest in Malaysia. 

## 2. Results

### 2.1. Isolation and Host Range Analysis of Phages

Using the primary enrichment method, a total of 13 phages (named PRF-SP1 to PRF-SP13) were isolated against different *Salmonella* serovars from the Penang National Park, Malaysia; four were isolated from water samples, while the other nine were isolated from soil samples. The details of the isolation source and their multidrug-resistant (MDR) hosts are provided in Table S1. We could not isolate any phages against the *Salmonella* Typhimurium serovar (Table S1). In terms of plaque morphology, all of the phages produced clear plaques (2–3 mm in diameter) on their host strains, suggesting that they are lytic in nature.

A spot test was performed to determine the host range of 13 phage isolates against 14 bacterial strains. The results indicated that two phages, PRF-SP1 and PRF-SP3, had a broad host range, where they were able to lyse all of the six tested *Salmonella* serovars and a clinical *E. coli* strain (Table 1). Phage PRF-SP4, on the other hand, was able to clearly lyse six *Salmonella* serovars, while PRF-SP5 was able to lyse at least five of them. The remaining phages had a relatively narrow host range, where they were only able to lyse fewer than four *Salmonella* serovars. Subsequently, phages with broad host ranges (PRF-SP1, PRF-SP3, PRF-SP4, and PRF-SP5) were chosen for further characterization. 

### 2.2. Phenotypic Characteristics of Phages 

**Figure 1:**
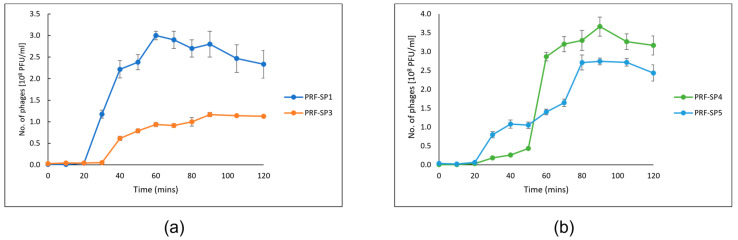
One-step growth curve. (**a**) Growth profile of phage PRF-SP1 and PRF-SP3 against *Salmonella* Paratyphi A CS/SPA18. (**b**) Growth profile of phage PRF-SP4 and PRF-SP5 against *Salmonella* Paratyphi B CS/SPB18.

The optimal multiplicity of infection (MOI) of PRF-SP1 and PRF-SP3 was 0.1, while phages PRF-SP4 and PRF-SP5 had an MOI of 0.01. Following this, the one-step growth of the phages was conducted with their respective host strain that was used for their initial enrichment. The growth profiles of the phages exhibited the three main phases; latent, rise, and plateau phase, as observed in Figure 1. 

**Figure 2:**
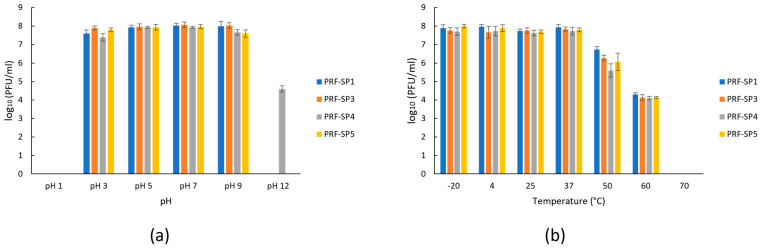
Stability profiles of phages over a range of pH and temperatures. (**a**) pH stability profile, (**b**) Thermal stability profile. Error bars indicate standard deviation among triplicate samples.

The growth characteristics of phages PRF-SP1 and PRF-SP3 were determined using *Salmonella* Paratyphi A CS/SPA18 as the host (Figure 1a). PRF-SP1 had a latent period of 20 min, with a burst size of 75 ± 8 PFU/cell, while PRF-SP3 exhibited a smaller burst size of 22 ± 4 PFU/cell and a longer latent period of 30 min. The growth profiles of phages PRF-SP4 and PRF-SP5, on the other hand, were completed using *Salmonella* Paratyphi B CS/SPB18 as their host culture (Figure 1b). Both of these phages had a latent period of 20 min, but their burst sizes varied considerably, as PRF-SP4 had a burst size of 120 ± 6 PFU/cell, while phage PRF-SP5 had a burst size of 42 ± 6 PFU/cell. As studies showed that the variation in the latent period and the burst sizes of different phages can be attributed to the different host cells being infected [21,22], the growth characteristics of all these four phages cannot be directly compared with each other. All of the phages were stable between pH 3 and 9 after 18 h of incubation (Figure 2a). The titer of the phages declined when they were exposed to more extreme pH values, of either less than 3 or more than 9. Over 0.02% of phage PRF-SP4 were viable at pH 12. The phages were also found to be actively stable between −20 °C and 50 °C, although there was a slight reduction in titer at 50 °C and 60 °C (Figure 2b). No phages were detectable at 70 °C after one hour of incubation. 

**Figure 3:**
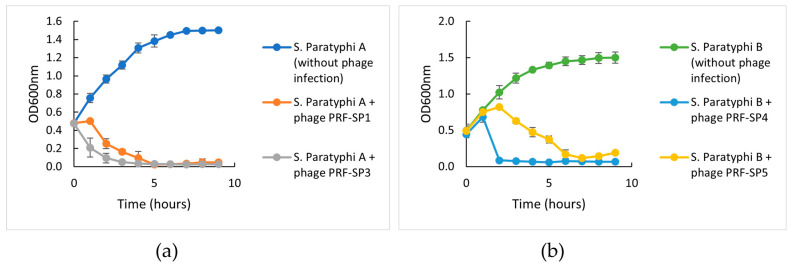
Bacterial challenge test of phages against their respective host *Salmonella* serovars. (**a**) Bacterial challenge test of phage PRF-SP1 and PRF-SP3 against *S*. Paratyphi A. *S*. Paratyphi A without phage infection was used as control. (**b**) Bacterial challenge test of phage PRF-SP4 and PRF-SP5 against *S*. Paratyphi B. *S*. Paratyphi B without phage infection was used as control. OD_600nm_ was measured via a spectrophotometer.

To investigate the ability of each phage to control *Salmonella* growth in vitro, a challenge test was performed with the addition of phage at their optimal MOI to the mid-exponential-phase host cultures. Similar to the one-step growth curve, a host challenge test was also performed with the *Salmonella* strain that was used for the initial enrichment of the respective phages. The challenge test of phages PRF-SP1 and PRF-SP3 was conducted against *S*. Paratyphi A (Figure 3a), while the challenge test of phages PRF-SP4 and PRF-SP5 was conducted against *S*. Paratyphi B (Figure 3b). Phages PRF-SP3 and PRF-SP4 were able to completely inhibit the growth of *Salmonella* at 2 h post-infection, while phages PRF-SP1 and PRF-SP5 caused lysis after 4 and 6 h, respectively. Host *Salmonella* cultures without phage infection, on the other hand, grew actively. Three of the phages, PRF-SP1, PRF-SP3, and PRF-SP4, were found to have a stable and high degree of lytic activity after a prolonged incubation of 9 h. In contrast, the growth of phage insensitive mutant bacteria was observed at the 8th hour, with the infection of phage PRF-SP5 (Figure 3b). 

**Figure 4:**
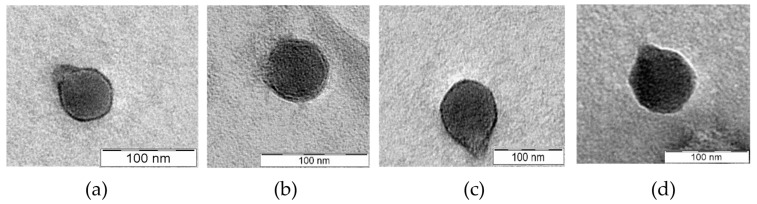
Electron microscopy images of negatively stained phage particles. Scale bar represents 100 nm. (**a**) PRF-SP1, (**b**) PRF-SP3, (**c**) PRF-SP4, (**d**) PRF-SP5.

TEM analysis of the four *Salmonella* phages (shown in Figure 4) revealed that all the phages have a podovirus morphology, with an isometric head (50 ± 2.1 nm–76 ± 2.2 nm) and a short tail (10 ± 2.5 nm–23 ± 2.2 nm). The Head diameters and tail lengths of each phage are provided in Table S2. 

### 2.3. Genome Characterization

All four phages (PRF-SP1, PRF-SP3, PRF-SP4, and PRF-SP5) were found to have linear dsDNA. The genome sizes of the phages ranged between 39,732 bp and 39,849 bp in length, and encoded 49 CDS, with a GC content of ~50% (Table S3). As in many previously annotated phage genomes, only a limited number of protein functions could be predicted, with 53% of the ORFs being annotated as hypothetical proteins with no known functions. The predicted ORFs encode genes mainly for structure (major capsid protein, scaffold protein, and tail tubular protein), packaging (portal protein and large terminase subunit), infection (tail spike protein), and host lysis (endolysin and spanin). Additionally, no tRNAs were found in any of these phages. The phage genomes also did not contain lysogeny-related modules encoding integrase, AMR genes, or virulence factors, suggesting that these phages might be safe for use in the biocontrol of *Salmonella.* However, because most of the ORFs were annotated as hypothetical proteins, a further investigation of the roles of these ORFs are needed to confirm that the phages are free from toxic or harmful contents. 

### 2.4. Comparative Genomic Analysis of Phages 

**Figure 5:**
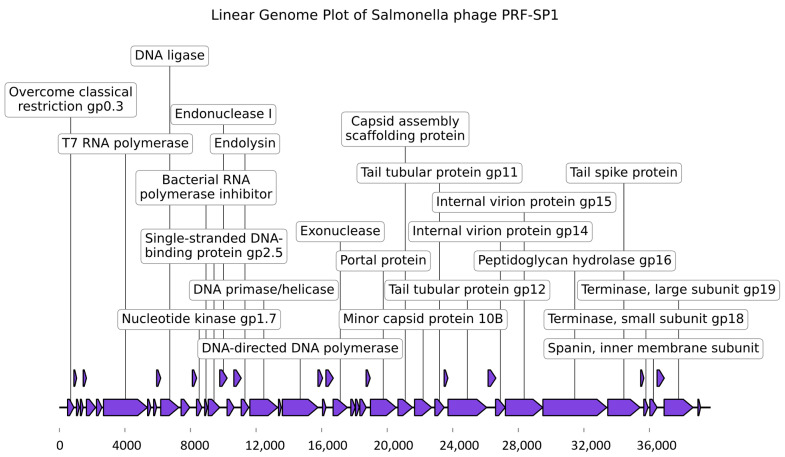
Linear genome map of Salmonella Phage PRF-SP1 using the tool Linear Genome Plot [26]. Out of 49 CDSs predicted, 23 CDSs encoded for functional proteins, as shown in the genome map.

All four phages were genetically similar to each other, with ~99% sequence similarity between them, as determined via EZBiocloud [23]. This is quite interesting, as they were isolated from different sources and different hosts (Table S1). A BLASTn [24] analysis against the NCBI NT database revealed that all of the newly isolated *Salmonella* phages in this study had a ~90% sequence similarity with their closest hits. Based on the recommendations by the International Committee on Taxonomy of Viruses (ICTV), these phages are classified into a new species, as they have <95% sequence similarity with the top hits from the NCBI database. Since there is an average of >70% sequence similarity between the newly isolated phages and the genomes from the database, they belong to the genus *Kayfunavirus* [25]. As the phages are genetically similar to each other, a representative genome map of phage PRF-SP1 is shown in Figure 5.

**Figure 6:**
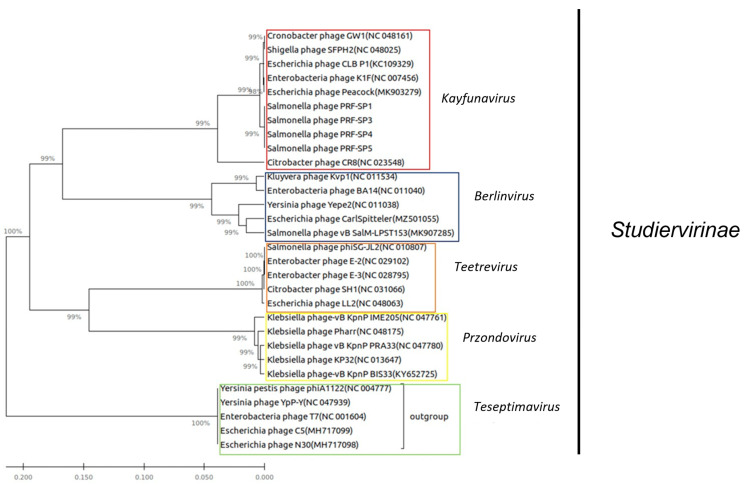
Maximum likelihood (ML) tree of the large terminase subunit of phages from the family *Autographviridae* and subfamily *Studiervirinae*. The midpoint rooted tree was inferred based on a ClustalW alignment of terminase large subunit (TerL) amino acid sequences from PRF-SP1, PRF-SP3, PRF-SP4, and PRF-SP5, and other closely related phage genomes available in GenBank. The bootstrap values (expressed as percentages) calculated from 1000 replicates are shown beside each node.

To further confirm the taxonomic classification of these *Salmonella* phages, the nucleotide sequence of the gene coding for the large terminase subunit of the phages was compared to other phages from the NCBI nt database. The alignment of the sequences was performed using ClustalW [27], while a phylogenetic tree was generated using MEGA7 [28], and the maximum likelihood method with 1000 bootstrap replicates. As shown in Figure 6, the clear phylogenetic distinction from other phages indicates that phages PRF-SP1, PRF-SP3, PRF-SP4, and PRF-SP5 are new members of the family *Autographviridae*, subfamily *Studiervirinae,* genus *Kayfunavirus*, and in the class of *Caudoviricetes*. The formation of a separate clade supports our findings that these phages belong to a new species, as they shared less than 95% ANI with other phages.

**Figure 7:**
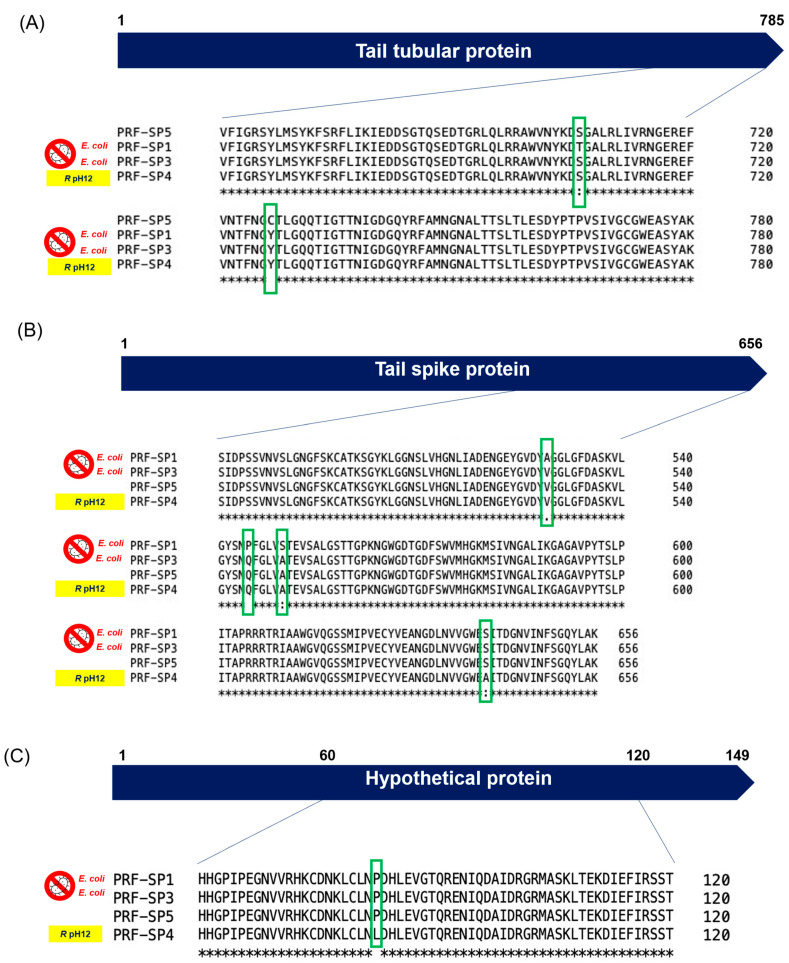
SNP analysis in phage protein sequences, and their phenotypic characteristics (PRF-SP4 is resistant to pH 12, while phages PRF-SP1 and PRF-SP3 lyse *E. coli*). Green boxes indicate the mutations in the proteins. (**A**) Tail tubular protein, (**B**) Tail spike protein, and (**C**) Hypothetical protein.

A closer look at the PRF-SP1, PRF-SP3, PRF-SP4, and PRF-SP5 genomes using dnadiff [29] revealed that the phages differed in three to seven Single Nucleotide Polymorphisms (SNPs) commonly found to be present in the phage–host interacting proteins such as the tail tubular protein gp12 and tail spike protein (Figure 7A,B). To a lesser extent, SNPs were also found in peptidoglycan hydrolase, T7 RNA polymerase, and in a hypothetical protein (Table S4). Around 90% (20/22) of the mutations were non-synonymous, and thus, they altered the amino acid sequences of the proteins in the respective phage genomes. Since 85% (17/20) of the SNPs were present in the tail-related proteins of the phages, a structural analysis of these proteins was conducted using Phyre^2^ [30]. The analysis revealed that all the four phages encoded a tail spike protein with two conserved regions: a pectate lyase-like catalytic domain with a beta-helix structure, and phage T7 tail fiber protein gp17. Phyre2 analysis was conducted for the hypothetical protein and tail tubular protein as well, but no conserved domains were predicted. One of the non-synonymous mutations was observed in a hypothetical protein (Figure 7C). We hypothesize that the non-synonymous mutation in the hypothetical protein may have influenced the size of the phage particle, as these phages had at least 50% difference in head size, despite being classified in the same family and having nearly identical genomes. 

## 3. Discussion

In the present study, 13 Salmonella phages (designated as PRF-SP1–PRF-SP13) were isolated from the water and soil samples collected from a protected, conserved rainforest in Malaysia, suggesting that the tropical rainforest is another potential source of novel phages. This is the first study that has shown that a tropical rainforest could also be a good source of phages against human pathogens such as *Salmonella*, apart from sewage and municipal waste treatment sites, which have always been of great interest for virologists to obtain lytic phages [31]. Given the vast diversity of micro-environments within rainforests, with thousands of plant species, the diversity of bacteria associated with these plants and co-occurring phages is also likely to be high. Expanding the work to study phages against other bacterial pathogens would be useful for enhancing our knowledge on the diversity of phages in Malaysian rainforest, which is poorly sampled/studied to date. 

A host range analysis of the 13 phages showed that 4 of the phages, PRF-SP1, PRF-SP3, PRF-SP4, and PRF-SP5, exhibited a broader host range compared to the other phages. For therapeutic applications, phages that are lytic and that have a broad host range activity are preferred over temperate and those with narrow lytic spectra [32]. This is due to the ability of temperate phages to transfer virulence and/or antibiotic resistance genes, whereas narrowly lytic phages are unable to infect a wide range of bacterial strains [33]. Four of these broad host range phages were further subjected to phenotypic characterization and comparative genomic analysis to better understand their biocontrol potentials.

The findings of the one-step growth curve experiment showed that phages PRF-SP1, PRF-SP4, and PRF-SP5 had shorter latent periods and larger burst sizes, compared to phage PRF-SP3. Phages with short latent periods and large burst sizes are desirable, as they are able to replicate more quickly, and new virion particles could be released faster [34], thereby efficiently controlling, or killing the bacterial host. However, the latent periods and burst sizes of different phages can be varied due to the differences in host cells, growth medium, pH, and temperature of incubation [21].

In addition, the isolated phages were tested for their stability when exposed to different external conditions, i.e., pH and temperature. As their ability to withstand a range of pH values and temperatures are vital for biocontrol applications, phages with stability characteristics that are too narrow might be ineffective during their application [35]. We observed that the phages showed a high degree of stability over a wide range of pH values, 3.0 to 9.0. The ability for these phages to retain a high degree of activity over a wide range of pH values allow them to be utilized in different food matrices [36]. Furthermore, one of the phages, PRF-SP4, was found to be slightly resistant to extreme alkaline conditions (pH 12). Apart from that, all four phages were stable at temperatures ranging from −20 °C to 50 °C, but no phages were detected when they were exposed to 70 °C. This might be due to the effect of increased temperature on the phage proteins. The overall results showed that the survivability of the phages is negatively affected by an increased exposure to high temperatures.

The host challenge test, on the other hand, revealed that all phages retained their lytic activity for the first seven hours, after which, phage-resistant bacterial mutants appeared in PRF-SP5-treated *Salmonella* culture. Unlike the rest of the phages, PRF-SP5 might not be a suitable candidate to be used individually as an antimicrobial agent against the *Salmonella* spp. The development of resistant mutants may be because of the behavior of host bacteria that forms proteins that block the recognition of phage receptor sites, or the digestion of phage genomes by the host strains [37]. As pathogens are known to have the ability to develop resistance to bacteriophages as early as six hours post-phage treatment [38], it is crucial to perform a proper screening of the phages prior to their use as biocontrol or therapeutic agents.

To better understand phage diversity, their suitability for downstream applications, and the differences observed in their phenotypic characteristics, phage genomes were sequenced and annotated. The morphological features observed in the TEM images, and the presence of a RNA polymerase-encoding gene in all their genomes confirmed that the phages are members of *Autographviridae* family, in the class of *Caudoviricetes* [39]. A further phylogenomic analysis revealed that these phages are new members of the genus *Kayfunavirus*. All four phages did not encode integrases, virulence-associated factors, or antimicrobial genes, as determined by PhageLeads [40], suggesting that these phages are safe for use in the biocontrol of *Salmonella*. Only lytic phages are preferred for phage therapy, as lysogenic phages have a high tendency to cause horizontal gene transfer between bacteria [41]. 

Further comparative genomic analysis showed that these phages share ~99% sequence similarity, where non-synonymous mutations were found to be present in the tail spike protein, tail tubular protein gp12, and a hypothetical protein (Figure 7). The tail spike protein of a phage is an important structure that affects the initial binding, while the tail tubular proteins often form a tubular structure surrounded by tail fibers [42]. In addition to their structural functions, a recent study suggested that the tail tubular proteins have a role in host recognition and attachment to its surface [43]. Mutations in these proteins can influence not only the injection of DNA into the host, but they could also affect the attachment kinetics of the phages to their host receptors, which might result in differences in their host specificities. So, it is reasonable to hypothesize that any mutations in tail-related proteins could play a major role in phage–bacteria interaction, leading to the phenotypic differences observed between the phages, even though they are genetically similar to each other. This could be clearly observed in the present study, where the phages have ~99% sequence similarity with each other, but they exhibit a different host lysis spectrum (Table 1) and pH resistance (Figure 2a). The differences in phage host lysis could be also due to the change in the methylation pattern of the host bacteria.

Phyre2 analysis revealed that the tail spike protein of all four phages encoded a pectate-lyase like catalytic domain. Pectate lyase is an important pectolytic enzyme that degrades galacturonic acid, a major component of bacterial polysaccharides [44]. We therefore hypothesize that the pectate lyase catalytic domain in the tail spike protein of these phages might be playing a role in aiding the degradation of host bacteria cell wall, and that it is essential for the initiation of a phage infection. This protein could be further used as an alternative antimicrobial agent, alone or in combination with phage, to enhance its effectiveness.

The findings of this study suggest that pristine environments, such as a conserved tropical rainforest, are an excellent source of novel phages against *Salmonella*. The phages isolated in this study can be used as potential alternatives to antibiotics, to reduce the prevalence and to better manage infections caused by MDR-*Salmonella* strains. Further studies are needed to evaluate the potential of these phages in the food industry, by extending the duration of the host challenge test, and by examining the biocontrol applications of these phages in *Salmonella*-infected animal models to assess their efficacy and safety. 

## 4. Materials and Methods

### 4.1. Bacterial Strains and Culture Conditions

Multidrug-resistant (MDR) *S. enterica* clinical strains belonging to six different serovars (*Salmonella* Paratyphi A CS/SPA18, *Salmonella* Paratyphi B CS/SPB18, *Salmonella* Paratyphi C CS/SPC18, *Salmonella* Enteritidis CS/SEN18, *Salmonella* Typhimurium CS/STYM18, and *Salmonella* Typhi B37239/20) were used as host strains to screen phages from the isolation sources. All bacterial strains were cultured from frozen glycerol stock onto Luria Bertani (LB) agar (1.5%) plates. Prior to the experiment, each strain was grown by inoculating a single colony from LB agar plates into LB broth, and incubating at 37 °C to obtain a fresh overnight culture.

### 4.2. Sample Collection

Water (500 mL) and two types of soil samples (~10 g) were collected from Penang National Park, a protected rainforest in Malaysia. One of the soil samples was dry and sandy, while the other was wet in texture. Following collection, samples were transported to the laboratory on ice, and stored at 4 °C, prior to phage isolation.

### 4.3. Isolation of Bacteriophages from Soil and Water Samples

Phage isolation from soil and water samples through enrichment was performed according to the method described by Twest and Kropinski (2009) [45], with some modifications. One gram of soil samples or 1 mL of water samples were added to a conical flask containing 10 mL of LB broth supplemented with 40 µL of 1 M calcium chloride (CaCl_2_). One hundred microliters of overnight host bacterial culture was also added into the conical flask. The mixture was then incubated at 37 °C for 24 h, with shaking at 100 rpm. After 24 h, the samples were centrifuged at 5776× *g* for 10 min, and the supernatants were filtered through 0.45- and 0.22-micron pore sized membrane filters. Ten microliters of the filtrate was spotted onto bacterial lawn culture, and the plate was incubated at 37 °C overnight. Following incubation, the plates were examined for lysis zones.

### 4.4. Purification of Bacteriophages 

All isolated phages were purified using the single plaque isolation method [13] through the double agar overlay technique. A single plaque was picked using a micropipette tip and transferred into 500 µL LB broth. This suspension was then subjected to a 10-fold serial dilution and double agar overlay assay. This purification process was repeated 5 to 7 times, until all plaque morphologies were consistent. 

### 4.5. Propagation and Determination of Phage Titer 

High titer phage stocks were prepared by inoculating 1 mL overnight host bacterial cultures with 100 µL of purified phage stock into 50 mL of LB (with 0.8 mM CaCl_2_), and incubating at 37 °C for 18–20 h. The amplified, purified phages were centrifuged at 5776× *g* for 10 min, and the supernatants were filtered through 0.45- and 0.22-micron pore sized membrane filters to remove bacterial contaminants. Phage titers were determined as plaque forming units (PFU/mL), using the double agar overlay technique. 

### 4.6. Host Range of Phages 

A host range study was performed according to Bao, (2015) [46] using 14 bacterial strains, including *Salmonella* Paratyphi A CS/SPA18, *Salmonella* Paratyphi B CS/SPB18, *Salmonella* Paratyphi C CS/SPC18, *Salmonella* Enteritidis CS/SEN18, *Salmonella* Typhimurium CS/STYM18, *Salmonella* Typhi B37239/20, *Salmonella* Typhi B41529/20, *Escherichia coli* O145:H28 strain RM12581, *Pseudomonas aeruginosa*, *Proteus mirabilis*, *Klebsiella pneumoniae*, *Vibrio cholerae* El Tor, and *Vibrio cholerae* O139 Bengal. The host ranges of the phages were determined via spot testing. A spot test was performed by spreading the bacterial cultures onto LB agar plates and adding 10 µL of phage lysates to the section of the agar plate. They were then allowed to dry, and they were incubated at 37 °C overnight. The next day, a zone of clearance/lysis were observed. The presence of a clear zone on the bacterial lawn indicated phage activity. The host specificity was also tested, using different dilutions of phages to determine the formation of clear lytic plaques. 

### 4.7. Determination of Multiplicity of Infection (MOI)

The host bacterial culture was grown in LB medium at 37 °C to log phase (10^8^ CFU/mL). One milliliter of bacterial cells was infected with five different dilutions (10^0^, 10^1^, 10^2^, 10^3^, and 10^4^) of phage lysate stock, with a titer of 10^10^ in a 10 mL 2× LB medium with the addition of 4 mM CaCl_2_. After incubation for 24 h at 37 °C with agitation at 100 rpm, the phage titers of these lysates were quantified as PFU/mL. The MOI resulting in the highest phage titer was determined to be the optimal MOI of the respective phages.

### 4.8. One-Step Growth Curve 

Burst sizes and latent periods of selected phages were determined via a one-step growth experiment according to a previous method [35]. Briefly, a bacteria phage suspension was prepared by mixing 100 µL of phage lysate with 900 µL of log phase host bacterial culture (10^8^ CFU/mL), according to the optimal MOI of the phage, and placed in a 37 °C incubator shaker for 10 min at 150 rpm. After 10 min of incubation, 100 µL of the mixture was transferred to a tube containing 9.9 mL LB broth (labelled 10^−2^), and the sample was further diluted to a dilution factor of 10^−6^. Tubes containing dilutions 10^−2^, 10^−4^, and 10^−6^ were then placed in a 37 °C incubator shaker for 2 h at 150 rpm. The phage titer was determined every 10 min using a double agar overlay method.

### 4.9. Transmission Electron Microscopy

High titer stocks of purified phages were prepared based on the method described by Jakočiūnė and Moodley (2018) [47]. Two milliliters of the phage stocks (10^10^) were centrifuged at 10,000 rpm for 1 h. Supernatants were carefully removed using a micropipette without disturbing the pellet. The pellet was washed twice in 0.1 M ammonium acetate (pH 7.0) and re-suspended in 200 µL of SM buffer. 

Using transmission electron microscopy (TEM), the morphologies of phages were examined via a single negative staining method. A drop of phage suspension was placed on carbon-coated formvar grids and allowed to stand for 3 min. The suspension was stained with 1% (*w*/*v*) uranyl acetate for 5 min, and the excess fluid was drawn off with filter paper. The grids were allowed to air dry, and then they were examined with a LEO 912AB Energy Filter TEM at 100 kV. The heads and tails of 5 individual phage particles were measured, and the average value was calculated. 

### 4.10. Phage Stability at Different Temperatures and pH Values 

Temperature and the pH stability of the phages were determined using the method proposed by Jamal et al. (2015) [38], with some modifications. The stability of the phages was tested in preheated sterile LB medium at different temperatures (−20 °C, 4 °C, 25 °C, 37 °C, 50 °C, and 65 °C) for 1 h. For the pH stability, bacteriophage suspensions were incubated at different pH ranges (1, 3, 5, 7, 9, and 12) for 18 h. After incubation, the titers of the phages in each sample were determined using the double agar overlay technique. Each treatment was performed in triplicates.

### 4.11. Host Challenge Test

The bacteriolytic activities of the phages were determined in vitro, as previously described by Amarillas et al. (2017) [48], with some modifications. Fifty milliliters of fresh TSB supplemented with 40µL of 1 M CaCl_2_ was inoculated with 1 mL of overnight cultures of *Salmonella* host strains and incubated at 37 °C, with shaking at 180 rpm, until the OD_600_ reached 0.4 (10^8^ CFU/mL). Thereafter, the phage lysate was added according to its optimal MOI. The bacterial growth was then monitored via turbidity measurements at OD_600_ nm every 1 h for 9 h. All of the experiments were performed in triplicate.

### 4.12. Genome Sequencing and Comparative Analysis

Phage DNA extraction was performed using CTAB-based buffer for lysis, while the further purification of DNA was performed using the phenol–chloroform method, as described by Minas et al. (2011) [49]. The DNA library was constructed using the Nextera DNA Flex library preparation kit, and paired-end sequenced using the Illumina MiSeq platform, with read lengths of 2 × 300 bp. Low-quality reads were trimmed with Trimmomatic v0.39 [50], and the trimmed reads were subjected to de novo genome assembly, using SPAdes v3.15.3 with default settings [51]. The quality of the assembly was assessed with QUAST v5.02 [52]. The reads were then mapped back against the resulting contig using Bowtie2 v2.4.4 to determine the average coverage of each contig [53]. The manual genome reordering of the phages was performed against a reference genome, based on the method reported by Shen and Millard [54]. The assembled genomes were then annotated using Prokka v1.12, https://github.com/tseemann/prokka, (accessed on 16 June 2022) using the database specified for the Kingdom Viruses [55]. The genomes were subjected to BLASTn analysis against the NT database to identify their closely related genomes. The Average Nucleotide Identity (ANI) was calculated with EZBiocloud, https://www.ezbiocloud.net/tools/ani, (accessed on 12 July 2022) between the isolated phages, to understand how similar they are to each other. dnadiff v1.3 in mummer was then used to extract the single nucleotide polymorphisms (SNPs) between the genetically similar phage genomes [29]. The phages were then screened for the presence of virulence and antibiotic resistance genes by subjecting them to PhageLeads, ResFinder v4.1, and Virulence Finder v2.0 [40,56]. The complete genomes of 15 phages sharing at least 85% identity and 85% query coverage were obtained from the NCBI database, to be used as reference genomes for further analysis. A comparative analysis of the large terminase subunit gene was performed with CLUSTALW multiple sequence alignment, and a phylogenetic tree was generated with MEGAX, using the Maximum likelihood method. 

## Figures and Tables

**Table 1:** Host ranges of the phages. ++ = Clear lysis, + = Turbid lysis, − = No lysis.

Bacteria/Phage	PRF-SP1	PRF-SP2	PRF-SP3	PRF-SP4	PRF-SP5	PRF-SP6	PRF-SP7	PRF-SP8	PRF-SP9	PRF-SP10	PRF-SP11	PRF-SP12	PRF-SP13
*S*. Paratyphi A CS/SPA18	++	++	++	+	+	+	++	++	++	++	++	−	−
*S*. Paratyphi B CS/SPB18	++	−	++	++	++	−	−	−	−	−	−	−	−
*S*. Paratyphi C CS/SPC18	++	++	++	++	++	++	++	++	++	++	++	−	−
*S*. Typhi B37239/20	++	++	++	++	++	++	++	++	++	++	++	−	−
*S*. Typhi B41529/20	++	++	++	++	++	++	++	++	++	++	++	−	−
*S*. Typhimurium CS/STYM18	++	−	++	++	+	−	−	−	−	−	−	−	−
*S*. Enteritidis CS/SEN18	++	−	++	++	++	−	−	−	−	−	−	++	++
*E. coli* O145:H28 strain RM12581	++	−	++	−	−	−	−	−	−	−	−	−	−
*Pseudomonas aeruginosa*	−	−	−	−	−	−	−	−	−	−	−	−	−
*Proteus mirabilis*	−	−	−	−	−	−	−	−	−	−	−	−	−
*Klebsiella pneumoniae*	−	−	−	−	−	−	−	−	−	−	−	−	−
*Vibrio cholerae* El Tor	−	−	−	−	−	−	−	−	−	−	−	−	−
*Vibrio cholerae* O139 Bengal	−	−	−	−	−	−	−	−	−	−	−	−	−

## Data Availability

The complete genome sequences of the Salmonella phages PRF-SP1, PRF-SP3, PRF-SP4, and PRF-SP5 have been deposited in GenBank, under the accession numbers of MZ923531, OL539729, OL773676, and OL773677, respectively. The raw data of the genomes can be found at the BioProject, PRJNA 760259.

## Supplementary Materials

The supporting information can be downloaded at: https://www.mdpi.com/article/10.3390/ijms24043678/s1.
