# Supplementary material for: Phenotypic Characterization and Comparative Genomic Analysis of Novel Salmonella Bacteriophages Isolated from a Tropical Rainforest
Source: Int J Mol Sci. 2023 Feb 12;24(4):3678. doi: 10.3390/ijms24043678 (PMC9963771; doi:10.3390/ijms24043678)
Supplement: Supplementary file 1 [file ijms-24-03678-s001.zip › ijms-2121729-supplementary.pdf]

Table S1: Summary of isolated phages with their respective isolation host and source of isolation.

**Phage ID**	**Isolation host serotype**	**Isolation source**	**GPS coordinate**
PRF-SP1	*Salmonella* Paratyphi A	Dry sandy soil	5°27'43.2"N 100°11'24.0"E
PRF-SP2	*Salmonella* Paratyphi A	Wet soil	5°26'56.1"N 100°11'42.8"E
PRF-SP3	*Salmonella* Paratyphi A	Pond Water	5°26'49.3"N 100°11'39.5"E
PRF-SP4	*Salmonella* Paratyphi B	Wet soil	5°26'56.1"N 100°11'42.8"E
PRF-SP5	*Salmonella* Paratyphi B	Pond Water	5°26'49.3"N 100°11'39.5"E
PRF-SP6	*Salmonella* Paratyphi B	Dry sandy soil	5°27'43.2"N 100°11'24.0"E
PRF-SP7	*Salmonella* Paratyphi C	Dry sandy soil	5°27'43.2"N 100°11'24.0"E
PRF-SP8	*Salmonella* Paratyphi C	Wet soil	5°26'56.1"N 100°11'42.8"E
PRF-SP9	*Salmonella* Paratyphi C	Pond Water	5°26'49.3"N 100°11'39.5"E
PRF-SP10	*Salmonella* Typhi	Dry sandy soil	5°27'43.2"N 100°11'24.0"E
PRF-SP11	*Salmonella* Typhi	Wet soil	5°26'56.1"N 100°11'42.8"E
PRF-SP12	*Salmonella* Enteritidis	Wet soil	5°26'56.1"N 100°11'42.8"E
PRF-SP13	*Salmonella* Enteritidis	Pond Water	5°26'49.3"N 100°11'39.5"E

Table S2: Morphological features of phages PRF-SP1, PRF-SP3, PRF-SP4 and PRF-SP5

**Phage ID**	**Head Diameter (nm)**	**Tail Length (nm)**
PRF-SP1	58 ± 2.5	23 ± 2.2
PRF-SP3	50 ± 2.1	15 ± 2.2
PRF-SP4	75 ± 2.4	18 ± 2.1
PRF-SP5	76 ± 2.2	10 ± 2.5

Table S3: Summary of phage genome details

Phage	Genome size (bp)	Number of Coding Sequence	GC content (%)
PRF-SP1	39,732	49	50.26
PRF-SP3	39,849	49	50.28
PRF-SP4	39,829	49	50.24
PRF-SP5	39,849	49	50.28

Table S4: Single Nucleotide Polymorphism analysis between the newly isolated phages with each other. A: PRF-SP1(Ref) vs PRF-SP4(Qry); B: PRF-SP1(Ref) vs PRF-SP3(Qry); C: PRF-SP1(Ref) vs PRF-SP5(Qry); D: PRF-SP3(Ref) vs PRF-SP5(Qry); E: PRF-SP4(Ref) vs PRF-SP5(Qry); F: PRF-SP3(Ref) vs PRF-SP4(Qry). **Ref = Reference genome; Qry= Query

A.

<table>
<tr><th>Phage</th><th>Position</th><th>Change</th><th>Effect</th><th>Amino acid change</th><th>Predicted function</th></tr>
<tr><td rowspan="6">PRF-SP1(REF)
vs
PRF-SP4
(QRY)</td><td>10893</td><td>C -> T</td><td>Non- Synonymous</td><td>P 83 L</td><td>Hypothetical protein</td></tr>
<tr><td>25828</td><td>A -> T</td><td>Non- Synonymous</td><td>T 706 S</td><td>Tail tubular protein gp12</td></tr>
<tr><td>35041</td><td>C -> T</td><td>Non- Synonymous</td><td>A 529 V</td><td rowspan="4">Tail spike protein</td></tr>
<tr><td>35089</td><td>C -> A</td><td>Non- Synonymous</td><td>P 545 Q</td></tr>
<tr><td>35103</td><td>T -> G</td><td>Non- Synonymous</td><td>S 550 A</td></tr>
<tr><td>35373</td><td>T -> G</td><td>Non- Synonymous</td><td>S 640 A</td></tr>
</table>

B.

<table>
<tr><th>Phage</th><th>Position</th><th>Change</th><th>Effect</th><th>Amino acid change</th><th>Predicted function</th></tr>
<tr><td rowspan="4">PRF-SP1(REF)
vs
PRF-SP3(QRY)</td><td>25828</td><td>A -> G</td><td>Non- Synonymous</td><td>T 706 S</td><td>Tail tubular protein gp12</td></tr>
<tr><td>35041</td><td>C -> T</td><td>Non- Synonymous</td><td>A 529 V</td><td rowspan="3">Tail spike protein</td></tr>
<tr><td>35089</td><td>C -> A</td><td>Non- Synonymous</td><td>P 545 Q</td></tr>
<tr><td>35103</td><td>T -> G</td><td>Non- Synonymous</td><td>S 550 A</td></tr>
</table>

C.

<table>
<tr><th>Phage</th><th>Position</th><th>Change</th><th>Effect</th><th>Amino acid change</th><th>Predicted function</th></tr>
<tr><td rowspan="5">PRF-SP1
(REF)
vs
PRF-SP5
(QRY)</td><td>25828</td><td>A -> T</td><td>Non- Synonymous</td><td>T 706 S</td><td rowspan="2">Tail tubular protein gp12</td></tr>
<tr><td>25892</td><td>A -> G</td><td>Non- Synonymous</td><td>Y 727 C</td></tr>
<tr><td>35041</td><td>C -> T</td><td>Non- Synonymous</td><td>A 529 V</td><td rowspan="3">Tail spike protein</td></tr>
<tr><td>35089</td><td>C -> A</td><td>Non- Synonymous</td><td>P 545 Q</td></tr>
<tr><td>35103</td><td>T -> G</td><td>Non- Synonymous</td><td>S 550 A</td></tr>
</table>

D.

Phage	Position	Change	Effect	Amino acid change	Predicted function
PRF-SP3 (REF) vs PRF-SP5 (QRY)	26009	T -> C	Non- Synonymous	Y 727 C	Tail tubular protein gp12

E.

Phage	Position	Change	Effect	Amino acid change	Predicted function
PRF-SP4 (REF) vs PRF-SP5 (QRY)	10990	T -> C	Non- Synonymous	P 83 L	Hypothetical protein
	33411	A -> G	Synonymous	-	Peptidoglycan hydrolase
	3063	G -> T	Synonymous	-	T7 RNA Polymerase
	35470	T -> G	Non- Synonymous	A 640 S	Tail spike protein

F.

Phage	Position	Change	Effect	Amino acid change	Predicted function
PRF-SP3 (REF) vs PRF-SP4 (QRY)	35490	A -> C	Non- Synonymous	S 640 A	Tail spike protein
	11010	G -> A	Non- Synonymous	P 83 L	Hypothetical protein
